# X-ray microbeam measurements with a high resolution scintillator fibre-optic dosimeter

**DOI:** 10.1038/s41598-017-12697-6

**Published:** 2017-09-29

**Authors:** James Archer, Enbang Li, Marco Petasecca, Andrew Dipuglia, Matthew Cameron, Andrew Stevenson, Chris Hall, Daniel Hausermann, Anatoly Rosenfeld, Michael Lerch

**Affiliations:** 1 Centre for Medical Radiation Physics, University of Wollongong, Wollongong, NSW 2522 Australia; 2 Illawarra Health and Medical Research Institute, University of Wollongong, Wollongong, NSW 2522 Australia; 3 Imaging and Medical Beam-Line, Australian Synchrotron, VIC, 3168 Australia

## Abstract

Synchrotron microbeam radiation therapy is a novel external beam therapy under investigation, that uses highly brilliant synchrotron x-rays in microbeams 50 *μ*m width, with separation of 400 *μ*m, as implemented here. Due to the fine spatial fractionation dosimetry of these beams is a challenging and complicated problem. In this proof-of-concept work, we present a fibre optic dosimeter that uses plastic scintillator as the radiation conversion material. We claim an ideal one-dimensional resolution of 50 *μ*m. Using plastic scintillator and fibre optic makes this dosimeter water-equivalent, a very desirable dosimetric property. The dosimeter was tested at the Australian Synchrotron, on the Imaging and Medical Beam-Line. The individual microbeams were able to be resolved and the peak-to-valley dose ratio and the full width at half maximum of the microbeams was measured. These results are compared to a semiconductor strip detector of the same spatial resolution. A percent depth dose was measured and compared to data acquired by an ionisation chamber. The results presented demonstrate significant steps towards the development of an optical dosimeter with the potential to be applied in quality assurance of microbeam radiation therapy, which is vital if clinical trials are to be performed on human patients.

## Introduction

**Figure 1:**
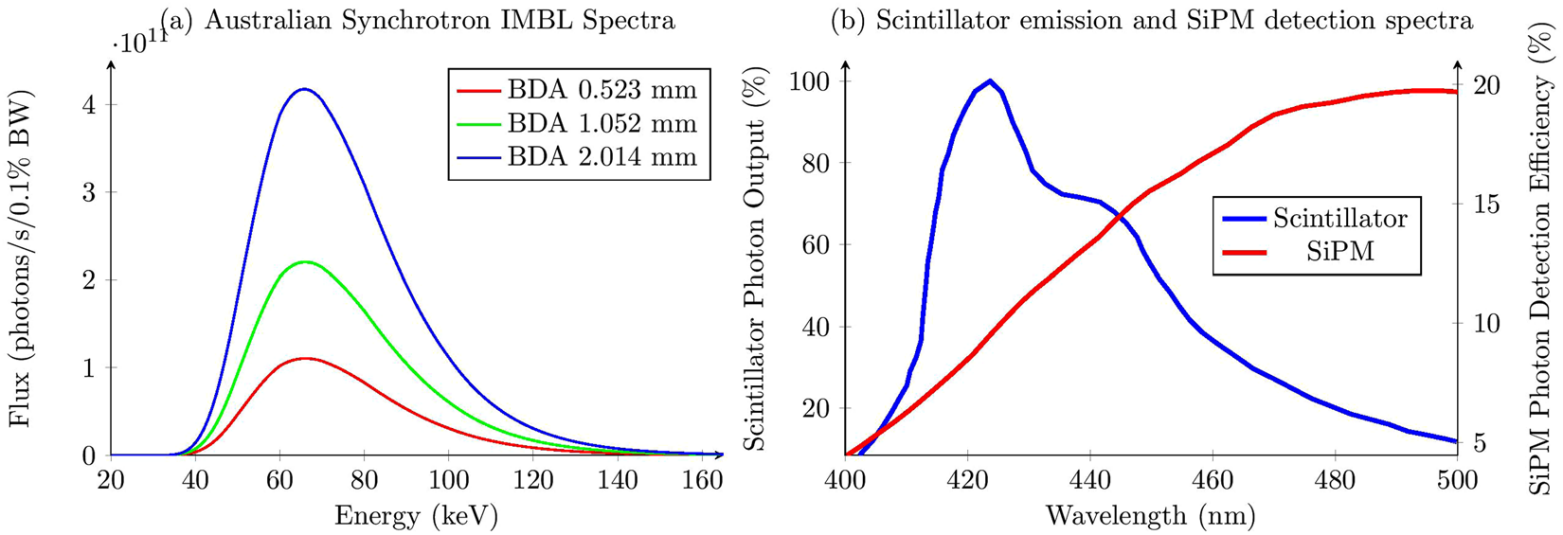
(**a**) The IMBL x-ray spectra calculated numerically using *spec.exe* by Stevenson *et al*.^1^, shown for the three beam defining aperture (BDA) heights. (**b**) BC-400 plastic scintillator emission spectrum and SensL SiPM photon detection efficiency.

Synchrotron microbeam radiation therapy (MRT) is a type of external beam radiation therapy that comprises of highly collimated planes of 70 keV median energy x-rays^1^ (see Fig. 1(a)) of typical thickness 50 *μ*m and separation 400 *μ*m. MRT has proposed applications in the treatment of brain tumours in children where conventional radiation therapies have a high risk to nervous system development. Animal studies have revealed the high potential of this new type of therapy^2–5^, and with the emergence of compact synchrotron technologies^6,7^ MRT has the potential to become widely available in the future. Two of the most important quantities to measure for quality assurance of MRT are the dose rates in the middle of the microbeams (the peaks) and the dose rate between the microbeams (the valleys)^8^. These quantities are important for characterising the radiobiological effect on the tissue to be treated in the peaks^9^ and the tissue to be spared in the valleys, which receives dose via Compton scattered electrons^10,11^. In addition the peak-to-valley dose ratio (PVDR) has major implications for the efficacy of the MRT. It is therefore necessary to have dosimeters with high enough resolution to measure these quantities. Dosimetry methods currently exist that have the required resolution for this purpose. Radiochromic film is commonly used for this purpose but take several hours to develop and process, and can be inaccurate by up to 15%^12^. Semiconductor devices such as metal-oxide semiconductor field-effect transistors (MOSFET)^13^ and silicon strip detectors (SSD)^14,15^ have excellent resolution and can resolve microbeams. Silica core optical fibers can also be doped (with molecules of hydroxide or fluoride) to produce light under irradiation. They have been demonstrated to show radiation hardness, with an approximately 25% increase in light absorbance after 1000 Gy dose^16^. However a major disadvantage of these dosimeters, as well as nearly all conventional dosimeters, is that they lack water-equivalence, meaning the dose measured by the dosimeter must be converted to the equivalent dose deposited in water, which can be a complicated function of radiation type and energy. This is necessary for clinical applications as the radiation dose deposited in tissue is the primary quantity of concern, and a water-equivalent dosimeter measures this dose.

Scintillators are one of the older radiation detectors^17^ but their application to high-resolution dosimetry has not been explored in great depth. A crystal scintillator dosimeter, which demonstrated a resolution of 100 *μ*m has been described by Belley *et al*.^18^. However as the scintillator is crystalline, it is not water-equivalent and so must be calibrated against an ionisation chamber, is only valid at calibrated energies, when the crystal is completely inside the microbeam. This means any measurements in the edges of microbeams may not result in the correct dose, and calculations of microbeam width may not be accurate.

The dosimeter presented in this work uses a BC-400 plastic scintillator optically coupled to an Eska CK-40 plastic optical fiber and coated in BC-620 reflective paint. The scintillator is the radiation conversion material, converting the energy of the x-ray secondary electrons to visible light (with peak light output at 423 nm (see Fig. 1(b)), and an efficiency of 11,300 photons per absorbed MeV), which is captured by the optical fiber. In plastic scintillators the radiation conversion occurs as the secondary electrons (generated via Compton scattering and ionisation) pass their kinetic energy to the electron orbitals around the organic molecules of the scintillator. The de-excitation of these orbitals emit photons in the visible spectrum. This design of dosimeter was originally developed by Beddar *et al* ^19,20^. for photon beams. In these articles the authors demonstrate that this dosimeter is highly water-equivalent, has excellent dose linearity and radiation hardness (2.8% response decrease for 10 kGy accumulated dose), and is energy independent. It also shows only very small changes in response under temperature and pressure variations. These qualities make it a very suitable dosimeter. In this work we improve upon this design by increasing the resolution of the probe from centimeters to 50 micrometers, allowing individual microbeams to be resolved.

There are further key characteristics that make this dosimeter design stand out from conventional dosimeters. There are only passive components in the radiation field, namely the scintillator and any fiber optic exposed to the field. All the electronic measurements are done out of the field, the main component being the photodetector which can be operated a safe distance from the radiation field. The components of the probe are also relatively inexpensive, the most expensive part of the design is the photodetector which, again, is safe from any radiation damage.

The major component reducing the water-equivalence of the probe is the BC-620 reflective paint. The paint contains 40% (by mass) TiO _2_ and so the titanium atoms scatter more radiation than lower Z materials, and increase the dose deposited compared to the amount that would be deposited in water or tissue.

Because this dosimeter is an optical system, there is no electronic interference. The main sources of unwanted signal are Cherenkov radiation and fluorescence generated in the fiber optic. Cherenkov radiation is generated by charged particles moving through a medium faster than the speed of light in that medium. For the refractive indexes used in this dosimeter, the minimum electron energy required to produce Cherenkov radiation is calculated to be approximately 175 keV^21^. The x-ray microbeams generated at the Australian Synchrotron, in the present case, have a peak flux at about 66 keV (this depends on the beam defining aperture (BDA) used, but the average photon energy remains 66 keV) and the percentage of photons above 175 keV energy is approximately 0.05%, meaning the amount of Cherenkov radiation noise in this system will not contribute greatly to the optical signal. Fluorescence in the fiber optic will therefore be the main contributor to any unwanted signal. The physical mechanism of fluorescence is the same as scintillation, but the intensity is much less per unit incident energy.

## Materials and Methods

**Figure 2:**
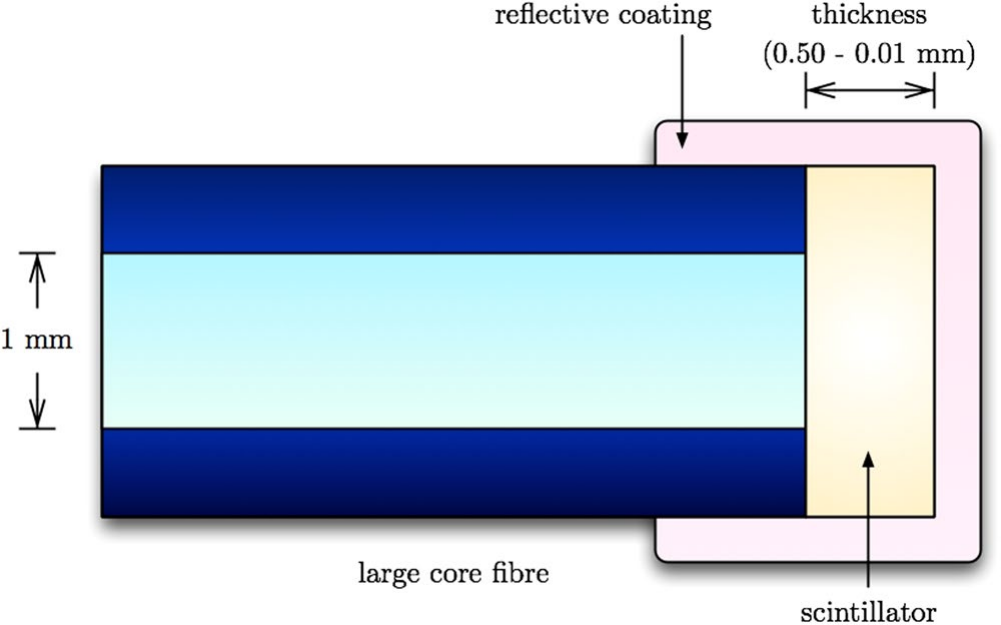
Scintillator sheet or film coupled to large core fiber (not to scale). Modified from Archer *et al*.^22^. For maximum one dimension resolution, the radiation beam must be incident perpendicular to the fibre axis (vertical in the image).

A diagram of the probe is shown in Fig. 2. The scintillator and fiber have a diameter of 1 mm, and the scintillator length is 50 *μ*m. This length defines the optimal one-dimensional resolution of the dosimeter, highest along the optical fiber axis. Perpendicular to the axis the resolution is 1 mm, the diameter of the scintillator. By scanning the probe along the fiber axis a lateral resolution of 50 *μ*m can be achieved over a depth of 1 mm. The design of this probe, with a 100 *μ*m resolution was demonstrated in a linear accelerator x-ray field in a previous work^22^.

The optical signal is measured using a SensL MiniSM Silicon Photomultiplier 10035 (SiPM). This SiPM has a peak sensitivity at 500 nm, with efficiency of 20%, and a typical dark count of 44 kHz (corresponding to a dark count, on average, every 20 *μ*s). The detection efficiency is compared to the BC-400 scintillator photon output spectrum in Fig. 1(b). The signal is digitised using a 14 bit Texas Instruments 64 channel analogue front end (AFE0064), using only a single channel. Custom software was used to acquire the digital signal. The software allowed the AFE0064 resolution scale, sample time (for an individual measurement), sample frequency and total run time to be altered. Increasing the sample time increases the signal collected from the SiPM, up to the sampling frequency, at the expense of temporal resolution. Due to the linearity and energy independence of the scintillator, the total light output is assumed to be proportional to the dose deposited in the sensitive volume.

**Figure 3:**
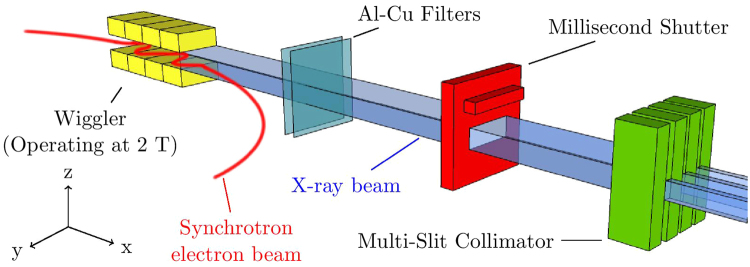
Diagram of the Australian Synchrotron Imaging and Medical Beam-Line components relevant to this work. The coordinate system used defines the beam direction as *x*, with microbeams fractionated in the *y* direction, with height in the *z* direction.

To demonstrate the ability of this dosimeter to resolve microbeams it was tested at the Australian Synchrotron on the Imaging and Medical Beam-Line (IMBL) (Illustrated in Fig. 3, where the coordinate system is also defined). Throughout the experiment the synchrotron beam current was 200 mA, and the IMBL wiggler insertion device was operated at 2.0 T. The beam can be spatially fractionated in the *y* direction into microbeams using a multi-slit collimator, without which a broadbeam can be used. The dimensions of the beam is then determined using a BDA of width 30 mm with available heights (restricting the beam in the *z* direction) of 2.014 mm, 1.052 mm and 0.532 mm.

The dosimeter was mounted on the Dynamic MRT stage in the beamline, which has motor controls for horizontal (*y* direction) and vertical (*z* direction) motion. The horizontal motor is a Parker MX80 S Ballscrew Drive with 50 mm range. The motor control allows speed to be controlled between 0.1 mm/s and 40 mm/s, which is within the operational ranges of the stage. The vertical motor is a Parker ZP200 Leadscrew Drive, which has a maximum scanning speed of 20 mm/s and a range of 25 mm. The reliability of this motor speed has been verified using an interferometer by IMBL scientists^1^.

A lateral scan of the beam profile was done to record the individual microbeams, allowing the PVDR to be calculated throughout the radiation field. The probe was oriented in edge-on mode, with the fibre axis aligned in the *y* axis: perpendicular to both the incident beam and the microbeams. The measurement was performed at a depth of 15 mm under solid water (Gammex 457 Solid Water^TM^), and the probe was scanned at a speed of 0.1 mm/s in the +*y* direction, allowing the entire profile to be measured in 400 s, including the response at the edge and outside of the field. Data points were sampled over a 200 *μ*s interval with a frequency of 2 kHz. The data acquired with the scintillation fibre optic dosimeter (FOD) was compared to an epitaxial silicon strip detector (SSD), also with a one-dimensional resolution of 50 *μ*m, developed by the Centre for Medical Radiation Physics (CMRP), scanned through the beam under identical conditions. The SSD is detailed in Fournier *et al* ^14^. and Lerch *et al*.^15^.

To remove any Cherenkov or fluorescence from the signal, we consider the amount of light generated per unit length of fibre, as this is the source of this unwanted signal. The signals should be identical both before and after the probe has been scanned through the field. However there is an increase in the signal when the probe has passed though the field, but the fibre remains exposed. A steady rise in detector response can also be seen in the valley regions, as more fibre is exposed. By using the background signals on either side of the profile, a linear fit can be made to the Cherenkov/fluorescence signal and subtracted off.

The depth dose response of the broad-beam field (without the collimator in place) was also measured by scanning vertically down the field (−*z*), averaging the response three times at each depth. The probe was mounted in the same orientation as the beam profile. The depth was changed by moving the probe in the + *x* direction and adding solid water between the beam and the probe. This was done at two scanning speeds, 5 mm/s and 10 mm/s, using each of the three BDAs. These results are compared to a PinPoint N31014 ionisation chamber under identical conditions.

## Results

**Figure 4:**
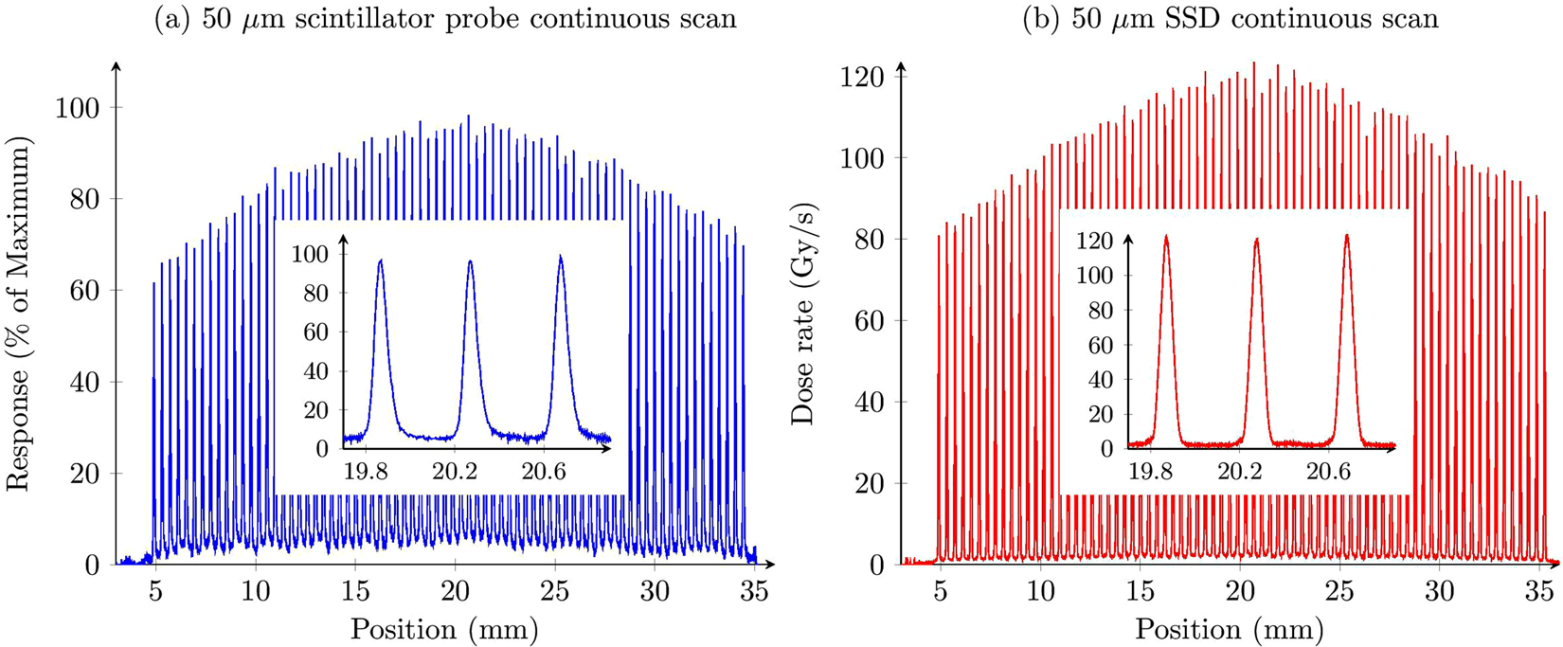
Intrinsic beam profile at 15 mm depth shown for (**a**) the scintillator probe, and (**b**) the SSD. Insets show the profile for the same three microbeams, close to the centre of the field.

**Figure 5:**
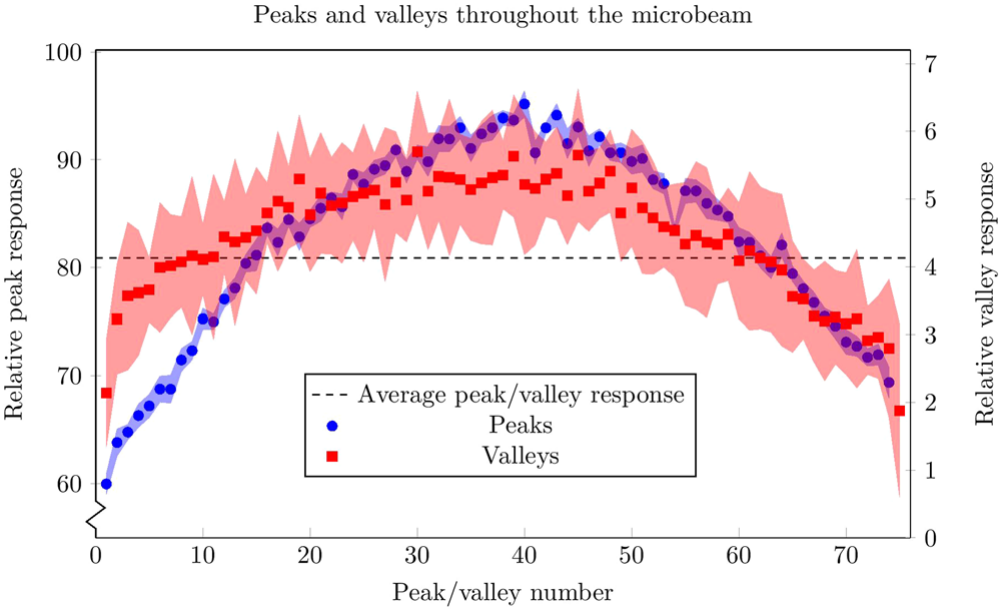
Heights of peaks and valleys throughout the beam. Vertical scales were chosen so the averages of both lie together. Error regions are the standard deviation of the average used to calculate the values. The maximum relative peak is not 100 as the normalisation was performed using the largest absolute value while each peak was found by averaging across the peak region to remove noise.

The microbeams are clearly resolved, which can be seen in Fig. 4(a), after Cherenkov/ fluorescence background subtraction. Using the detector response at both edges of the field, the background contribution was calculated to be 11.4 counts/mm and was subtracted off. The response was normalised to the maximum response value. A moving average of width 17 points was used to smooth the profile. This greatly reduced the noise in the signal, and the width was small enough to not reduce the peak responses. The inset of Fig. 4(a) shows three individual microbeams. There is a slight asymmetry apparent on the right side of each microbeam. The microbeams match up well with the SSD results in Fig. 4(b). The values of each peak and valley region are shown in Fig. 5. The peak values were determined by finding the position where the local maximum value lie, and taking the average over a 2.25 *μ*m region. The exact height of the peaks is obscured by noise in the data, and so averaging over this region gave a more robust value of the peak heights. The same was done for the valley regions, over a distance of 100 *μ*m to calculate the mean valley response. The error bars are the standard deviations of each average.

The average PVDR was calculated to be (19 ± 3). The average width (full width at half maximum) of the peaks was calculated to be (63 ± 2) *μ*m. For comparison, the SSD measured the PVDR to be (26.9 ± 1.4) and the FWHM to be (62.4 ± 0.9) *μ*m.

**Figure 6:**
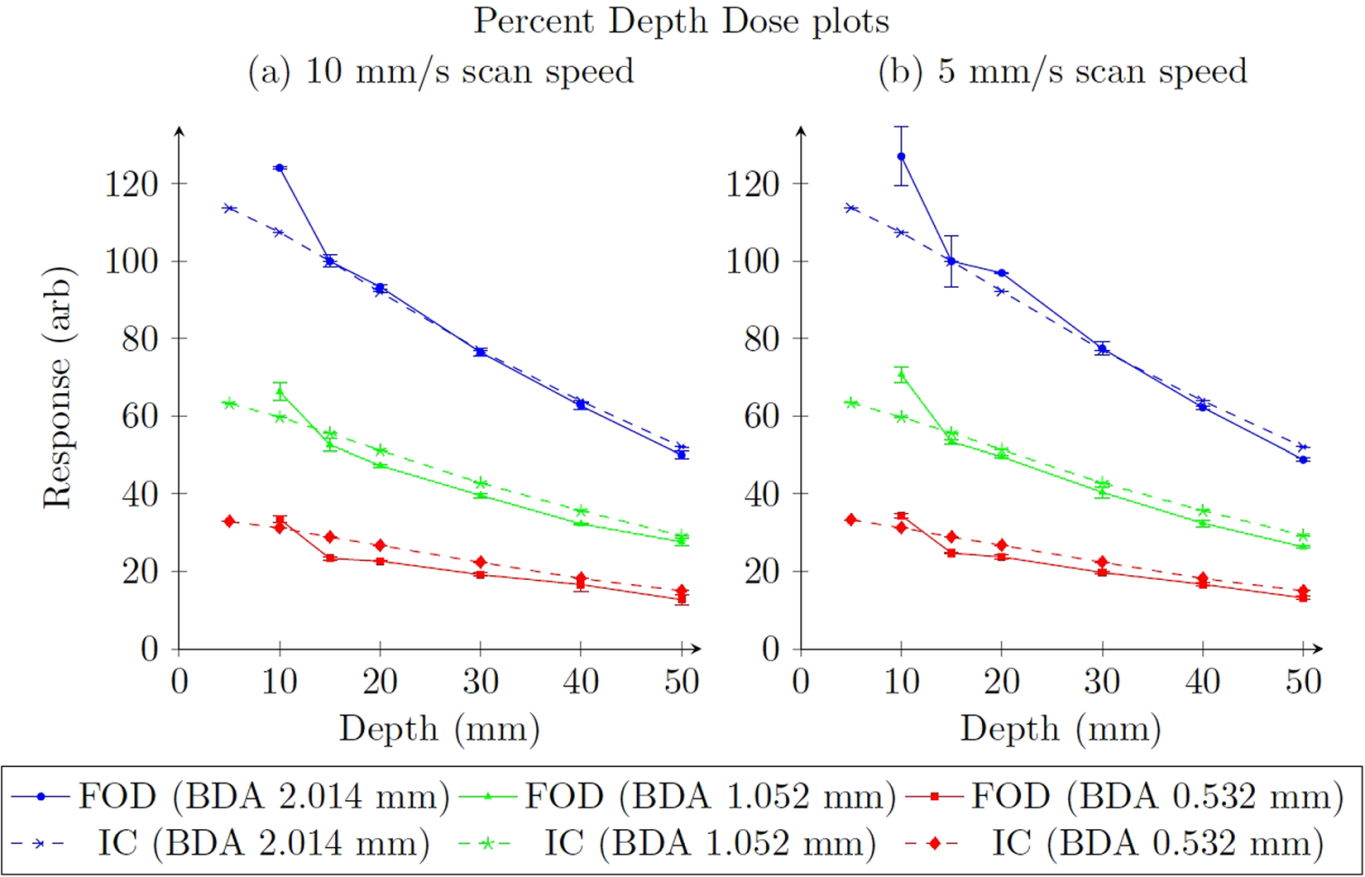
PDD plots at the two scanning speeds of the scintillator fibre optic dosimeter (FOD), with PinPoint N31014 ionisation chamber (IC) data under identical conditions. Both sets of data are normalised to the 2.014 mm BDA at 15 mm depth. Error bars are the standard deviation of three repeated measurements.

The results of the PDD scans are presented in Fig. 6. After normalisation to the 2.014 mm BDA at 15 mm depth, the 2.014 mm BDA results are in good agreement. The other BDAs slightly under-respond compared to ionisation chamber results. At 10 mm depth the dosimeter over-responds by approximately 20% when compared to the ionisation chamber. This is consistent across all BDAs and scan speeds.

## Discussion

The slight asymmetry apparent in the inset of the beam profile (Fig. 4) is due to the geometry of the scintillator probe. Two factors will contribute to this: firstly, if the thickness of the scintillator is not exactly 50 *μ*m across the 1mm diameter cross-section, the response may be different on either side of the scintillator. Secondly, if the fibre is not perfectly aligned perpendicular to the incident microbeam field then this will also create an asymmetry in the response. There is also an asymmetry present in the SSD profile, although it is less significant.

The convolution of a 50 *μ*m sensitive volume with a 50 *μ*m width microbeam will result in the measured FWHM being larger than the microbeam itself. The average FWHM measured with the scintillator probe is (63 ± 2) *μ*m, which agrees with result from the SSD of (62.4 ± 0.9) *μ*m, which verifies the resolution of the scintillator probe to be 50 *μ*m (the same as the SSD). The PVDR using the scintillator of (19 ± 3) is much lower than the SSD result of (26.9 ± 1.4). This can be explained by the higher response in the valley regions in the scintillator probe than the SSD.

The error bars in the valley response in Fig. 5 are high due to the low signal-to-noise ratio in the valleys, where the signal is very low. Possible methods for improvement include reducing the length of fibre used, which attenuates the scintillator light as it is transported. As is seen in Fig. 1(b) the overlap between the BC-400 emission spectrum and the SiPM photon detection efficiency is limited. Higher wavelength scintillators may provide a more favorable combination.

At the scanning speed of 0.1 mm/s and a sample time of 200 *μ*s the probe will move a distance of 0.02 *μ*m. Compared to the sensitive length along the fibre axis of 50 *μ*m the blurring caused by sampling while scanning is negligible. The scan speed of the beam profile measurement can be increased, as much more data was acquired than necessary. Increasing the scanning speed and decreasing the sampling frequency will greatly reduce the time taken to acquire the data as well as the quantity of the data.

The PDD results are of great interest, given the consistent and repeatable over-response at low depths. In a future experiment we will repeat these measurements, taking more values between zero depth and 20 mm to greater characterise this discrepancy.

Radiation damage to the detector was assumed to be negligible for this experiment, as the total dose the probe was exposed to during the PDD measurements was 2 Gy, (which was the experiment where the probe was exposed to the most dose). As was demonstrated by Beddar *et al* ^19^. their dosimeter (made from the same materials as the one in this work) saw less than a 3% decrease in responsiveness from 10 kGy dose, justifying this assumption.

An improved method for subtraction of Cherenkov and fluorescence background signal is to use an identical fibre running parallel to the probe, but without the scintillator. This provides a source of background light, but no scintillation light. For real time dosimetry in fields of high dose gradients (such as MRT) this method is not very practical, however we intend to measure the background signal separately in future works to compare to the method applied in this work to assess its validity.

The water-equivalence of this probe is reduced by the optical paint. As the paint is a diffuse reflector, the expected increase in light capture is not very large, and as the paint forms a layer on the order of 100 *μ*m thick, the compromise to water-equivalence may not be justified by the increased light capture. We intend to investigate the effect of replacing the paint layer with an aluminium coating. The advantages of this is twofold: aluminium is a lower Z number material than titanium, and the layer can be applied less than 1 *μ*m thick, reducing the interaction of the aluminium with the ionising radiation. The signal strength with no reflector is also an area to be explored.

The next step for this work is twofold: experimental and computational. We will fabricate a probe of higher spatial resolution, aiming for probes of 20 *μ*m and 10 *μ*m thickness, to test the limits of possible resolution of this probe design, while maintaining a practical level of sensitivity. Now that we have demonstrated the function of the dosimeter, the data collection methodology will be optimised. For example, the combination of 200 *μ*s sample time and 2 kHz sample rate provides much more data than necessary, increasing digital data processing time and memory. As the sample frequency corresponds to a distance of 50 nm (compared to a spatial resolution of 50 *μ*m), at 0.1 mm/s, increasing the scanning speed will allow a much quicker measurement of the intrinsic beam profile.

We will also perform a Geant4 Monte Carlo study to provide a comparison to the experimental results. This will allow validation of the PVDR and peak widths measured using this probe, and will allow the optimal scintillator thickness to be investigated.

## Conclusion

This work demonstrates the first water-equivalent plastic scintillator optical dosimeter applied to relative microbeam dosimetry, with the highest spatial resolution found in the literature of 50 *μ*m. The individual microbeams have been resolved, and important beam properties, such as the PVDR, have been calculated. The probe has been compared to both a SSD dosimeter and ionisation chamber and the results agree, with a few exceptions. This proof-of-concept work has demonstrated that is is possible to perform microbeam dosimetry with a plastic scintillator probe, and justifies the development of higher spatial resolution probes of this design. The use of an optical system overcomes challenges presented by using electronic dosimeters, and the simple and inexpensive nature of the probe makes this area of research very promising for the challenging area of clinical microbeam dosimetry.
